# Prediction models for post-induction hypotension in patients undergoing general anesthesia: a systematic review and meta-analysis

**DOI:** 10.3389/fmed.2026.1887058

**Published:** 2026-09-08

**Authors:** Lu Meng, Kanru Zhao, Long Shen, Yuelai Yang

**Affiliations:** Department of Nursing, Shanghai Ninth People’s Hospital, Shanghai Jiao Tong University School of Medicine, Shanghai, China

**Keywords:** general anesthesia, machine learning, meta analysis, post induction hypotension, prediction model, systematic review

## Abstract

**Background:**

Post-induction hypotension (PIH) is a frequent perioperative complication during general anesthesia and adversely affects patient outcomes. Although machine-learning techniques have been widely applied to develop PIH prediction models, the methodological quality and predictive performance of existing models lack systematic evaluation.

**Objective:**

To systematically review the predictive performance, methodological quality, and common predictors of PIH prediction models in patients undergoing general anesthesia, and to provide evidence for clinical practice and future model development.

**Methods:**

PubMed, Embase, Web of Science, Cochrane Library, CNKI, and Wanfang Data were searched from inception to April 2026 for studies developing or validating PIH prediction models. 2 reviewers independently screened studies, extracted data, and assessed risk of bias using the Prediction model Risk of Bias Assessment Tool (PROBAST). A random-effects meta-analysis was performed for the area under the receiver operating characteristic curve (AUC). Pooled odds ratios (ORs) for predictors appearing in at least 3 studies were calculated.

**Results:**

17 studies (50 prediction models, 40,864 patients) published between 2011 and 2026 were included. The AUC/C-statistics reported by the modeling groups ranged from 0.68 to 0.95, and those reported by the validation groups ranged from 0.654 to 0.893, only 1 model was validated on an external institutional dataset (AUC 0.654). The pooled AUC of the 17 optimal models was 0.81 (95% CI 0.77–0.85). Age (OR 1.031, 95% CI 1.016–1.046) and propofol dose (OR 1.357, 95% CI 1.046–1.667) were significant risk predictors. PROBAST assessment rated 15 studies (88.2%) as having high overall risk of bias and 2 studies (11.8%) as low risk; most concerns arose from the statistical analysis domain.

**Conclusion:**

Current PIH prediction models show good discriminative ability, but most have a high risk of bias and lack external validation. Future research should standardize the definition of PIH, improve predictor selection, and conduct multicenter external validation to facilitate clinical translation.

**Systematic review registration:**

CRD420261372822

## 1 Introduction

Post-induction hypotension (PIH) generally refers to a decline in blood pressure occurring within a specific time window from the start of general anesthesia induction to skin incision (1). Unlike intraoperative hypotension that may develop throughout surgery, PIH has a distinct pathophysiology-it occurs predominantly during the “hemodynamic vacuum period” when peak anesthetic effects have not yet been counteracted by surgical stimulation, representing a phase of pronounced perioperative circulatory fluctuation (2). A uniform definition of PIH is currently lacking, and the thresholds used vary considerably across studies. Commonly adopted definitions include mean arterial pressure (MAP) < 65 mmHg, a > 30% decrease in MAP from baseline, and systolic arterial pressure <90 mmHg (3). This definitional heterogeneity leads to widely variable reported incidences, ranging from 10 to 89% (3); generally, the higher the threshold selected, the higher the observed incidence of hypotension (4).

PIH is a major component of the broader burden of intraoperative hypotension and is significantly associated with multiple organ dysfunction, including myocardial injury (5), acute kidney injury (6), and stroke (7), as well as with increased postoperative mortality (8). The pathophysiology of PIH is multifactorial and incompletely understood. Preoperative fasting, bowel preparation, and other patient-related factors may lead to relative hypovolemia, reducing cardiovascular reserve before anesthesia induction (9). Anesthetic agents contribute through arterial vasodilation that decreases systemic vascular resistance, venous dilation that reduces venous return and cardiac preload, direct myocardial depression, and inhibition of the baroreflex that impairs compensatory responses to acute blood pressure changes (10–12). Additionally, the absence of surgical stimulation after induction may result in excessive anesthetic depth, further exacerbating hypotension. Patient-specific factors, such as diabetes mellitus and chronic hypertension, are associated with impaired autonomic nervous system regulation, manifesting as cardiac chronotropic incompetence and an inadequate response to hypoperfusion (13). Consequently, PIH often develops abruptly and unpredictably. During the induction period, anesthesia providers must manage multiple tasks simultaneously, and their divided attention may delay the initiation of corrective interventions.

The occurrence of PIH is influenced by multiple interacting factors, and the complex interactions among these factors cannot be adequately captured by traditional linear statistical methods. Although many prediction models for PIH have been developed, some studies still rely on univariate screening for variable selection, potentially omitting important predictors. Published systematic reviews of perioperative prediction models have mainly focused on topics such as intraoperative hypotension prediction (14). Given the rapid accumulation of research in this field and the varying methodological quality of available studies, this systematic review and meta-analysis aims to provide an evidence-based foundation for the development, validation, and clinical translation of high-quality PIH prediction models.

## 2 Methods

This review follows the PRISMA 2020 statement (15) and was registered with PROSPERO (CRD420261372822).

### 2.1 Eligibility criteria

Inclusion criteria: (1) patients aged ≥18 years undergoing general anesthesia; (2) studies developing, validating, or updating a prediction model for PIH; (3) The prediction window for PIH was explicitly defined as a specific time interval after anesthetic induction, which typically falls before skin incision; (4) cohort, case–control, or cross-sectional design; (5) at least one model performance metric reported (e.g., AUC, C-statistic, sensitivity, specificity); (6) English or Chinese language.

Exclusion criteria: (1) risk factor studies without a complete prediction model; (2) models predicting postoperative or intraoperative hypotension without extractable PIH data; (3) animal studies; (4) reviews, guidelines, conference abstracts, or case reports; (5) models with ≤2 predictors; (6) full text unavailable or incomplete data.

### 2.2 Search strategy

PubMed, Embase, Web of Science, Cochrane Library, CNKI, and Wanfang Data were searched from inception to April 2026 using a combination of MeSH terms and free words. Reference lists of included articles were also screened. The detailed search strategy is provided in Supplementary file 1.

### 2.3 Study selection and data extraction

Two reviewers independently screened titles/abstracts and then full texts. Disagreements were resolved by a third reviewer. Data extraction followed the CHARMS checklist (16) using a pre-designed form, covering: (1) study characteristics (first author, year, country, design, data source, population, surgery type); (2) model characteristics (modeling method, predictor selection, final predictors, presentation, missing data handling); (3) performance metrics (AUC with 95% CI, validation AUC, sensitivity, specificity, calibration method); (4) validation method (internal/external). In this review, internal validation refers to any validation method using data from the same institution or study population, including random split, cross-validation, bootstrap, and temporal validation (different time period, same institution). External validation is defined strictly as validation on data collected from a different institution or geographic location (non-homologous data). Two reviewers cross-checked the extracted data.

### 2.4 Risk of bias and applicability assessment

Two reviewers independently used PROBAST (17) to assess risk of bias and applicability. PROBAST covers four bias domains (participants, predictors, outcome, analysis) and three applicability domains. A study was rated as low overall risk of bias if all four bias domains were low; high risk if any domain was high; unclear otherwise. Applicability was rated similarly.

### 2.5 Statistical analysis

Analyses were performed with R 4.4.0. Meta-analysis of discrimination: We selected the best-performing model from each study (validation AUC prioritized over training AUC). A random-effects model was used to pool AUCs. Heterogeneity was assessed with *I^2^*. Sensitivity analysis excluded extreme AUC values. To further guard against overoptimistic estimates derived from training-set AUCs, we performed a supplementary sensitivity analysis that restricted the meta-analysis to studies reporting a validation AUC (either internal or external). For studies with multiple validation estimates (e.g., both temporal and random-split), the validation AUC with the most rigorous design (external > temporal > internal) was prioritized. Subgroup analyses were performed based on predefined categories: sample size (<1,000, 1,000–3,000, ≥3,000) to reflect small, medium, and large cohorts; event rate (<0.4, 0.4–0.6, ≥0.6) to represent low, moderate, and high incidence; and number of predictors (<10, ≥10) based on the median split; and modeling method (logistic regression/random forest/other). Meta-analysis of predictors: For predictors appearing in ≥3 studies, we pooled unadjusted ORs using a random-effects model.

## 3 Results

### 3.1 Study selection

**Figure 1:**
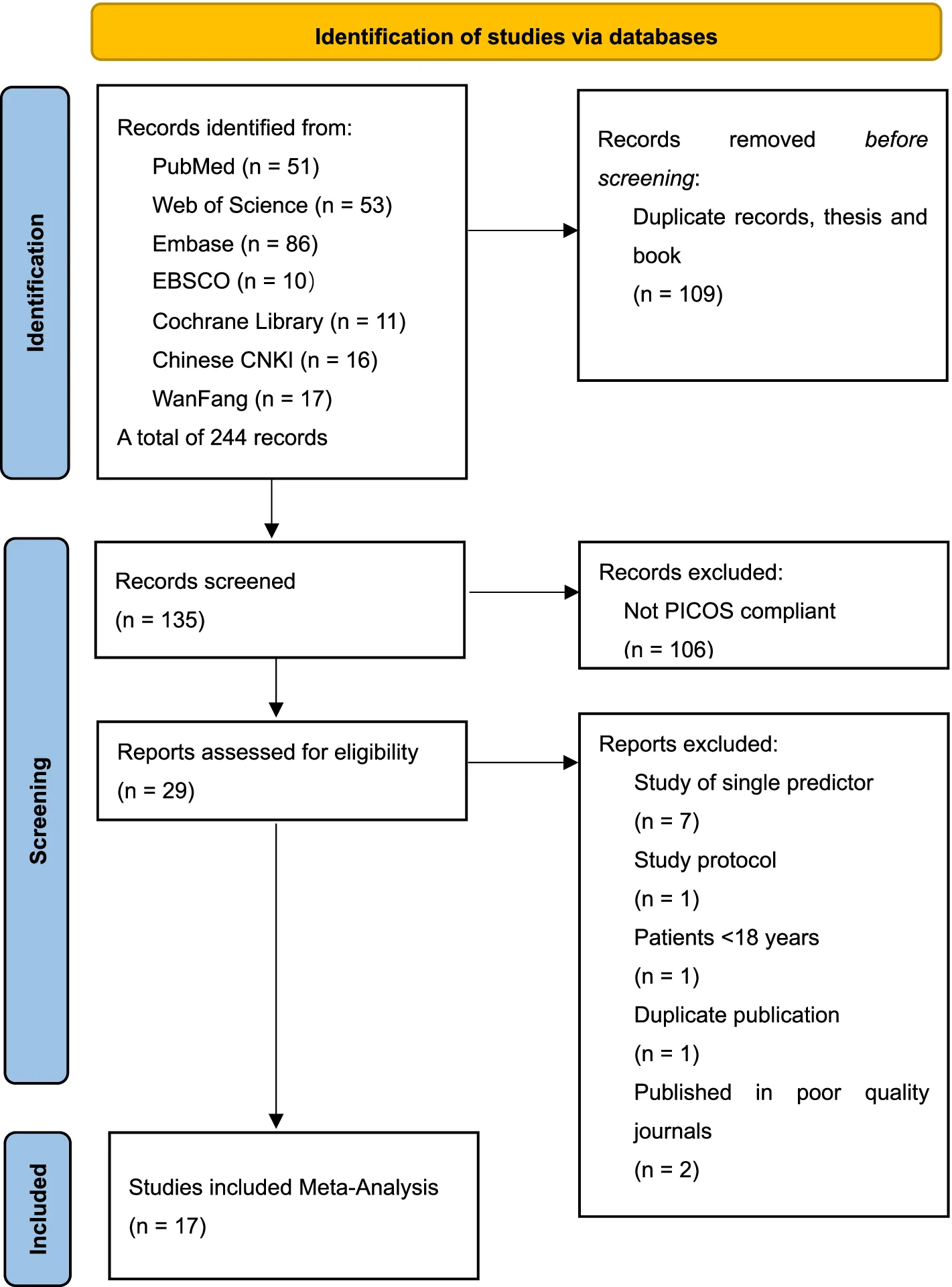
Flow chart summarizing study identification and selection.

A systematic literature search identified 244 records. After removing duplicates, 135 articles remained for title and abstract screening, of which 109 were excluded. The full texts of the remaining 29 articles were assessed for eligibility, and 17 studies met the inclusion criteria (Figure 1).

### 3.2 Study characteristics

The 17 studies were published between 2011 and 2026, encompassing a total of 40,864 patients undergoing general anesthesia. Among these studies, 15 employed a retrospective cohort design and two used a prospective cohort design. Geographically, ten studies were conducted in China, two in South Korea, two in Japan, and one each in Israel, Iran, and the Netherlands. Surgical procedures varied, including high-risk cardiac surgery, transcatheter aortic valve implantation, emergency acute type A aortic dissection, intermediate-risk procedures (e.g., laparoscopic cholecystectomy, colorectal cancer resection, urological surgery), and mixed surgical populations. Notably, the definition of PIH was heterogeneous across studies, with thresholds including an absolute MAP <55–65 mmHg, SBP < 90 mmHg, or >20–30% decline from baseline, or composite definitions requiring vasopressor use. Detailed study and patient characteristics are summarized in Tables 1, 2.

**Table 1:** Study characteristics of included prediction models.

Author/Year	Country	Design	Population	Data source	PIH definition (mmHg)	Prediction window	Model type
Lin 2011 (18)	Taiwan, China	Retrospective study	General intravenous anesthesia	Taipei Medical University Hospital	SBP < 90 or >30% drop	Induction to incision	Dev + Val
Kang 2020 (19)	South Korea	Retrospective study	Lap. cholecystectomy	Soonchunhyang Univ. Bucheon	SBP < 90 or MAP<65	Intubation to incision	Dev + CV
Li 2021 (20)	China	Retrospective study	Cardiac surgery	Second Affiliated Hospital of Hainan Medical University	SBP < 90 or MAP<65	First 10 min after induction	Dev + Val
Yoshimura 2022 (21)	Japan	Retrospective study	Non-cardiac	Ube Industries Central Hospital	MAP<55	Intubation to incision	Dev + Val
Zhao 2023 (22)	Netherlands	Retrospective study	General anesthesia	VitalDB	SBP < 75 or >30% drop	First 15 min after induction	Dev
Park 2024 (23)	South Korea	Retrospective study	Non-cardiac	VitalDB	MAP<65 for ≥6 s	First 20 min after induction	Dev
Noto 2024 (24)	Japan	Retrospective study	Transcatheter aortic valve implantation	Hirosaki University Hospital	SAP<90 or norepinephrine >6 μg/min	Induction to 20 min or incision	Dev + Val
Sheng 2024 (25)	China	Retrospective study	Colorectal cancer resection	Beijing Shijitan Hospital	MAP drop >20% from baseline	Induction to incision or 20 min	Dev + Val
Wang 2024 (26)	China	Retrospective study	Lap. cholecystectomy	Second Hospital of Anhui Medical University	MAP drop >30%	Induction to 20 min or incision	Dev + Val
Yang 2024 (27)	China	Retrospective study	Elderly non-cardiac	Zhongshan Hospital (Qingpu)	SBP drop >30% or MAP<60	Induction to intubation	Dev + Val
Hashemi 2025 (28)	Iran	Prospective cohort study	General anesthesia	Multi-center	MAP drop>20% or MAP<65 or SBP < 90	Next 10 min	Dev
Chen 2025 (29)	China	Retrospective study	General, thoracic, urologic, gynecologic	VitalDB	MAP<55	Induction to incision	Dev + Val
Sheng 2025 (30)	China	Retrospective study	Elective general anesthesia	Ningbo University Affiliated People’s Hospital	MAP<65	After induction	Dev
Bian 2025 (31)	China	Prospective cohort study	Non-cardiac	First Affiliated Hospital of Soochow University	MAP≤65 or drop>30%	First 15 min or incision	Dev + Val
Katsin 2025 (32)	Israel	Retrospective study	Non-obstetric general anesthesia	Sheba Medical Center	MAP<55	First 10 min after induction	Dev + Val
Xu 2026 (33)	China	Retrospective study	Elderly urologic	Ruijin Hospital	SBP < 90 or MAP<60 or vasopressor use	First 20 min after induction	Dev + Val
Zhu 2026 (34)	China	Retrospective study	Acute type A aortic dissection	Drum Tower Hospital	MAP drop≥30% or MAP<55	First 15 min or incision	Dev + Val

SBP, systolic blood pressure; MAP, mean arterial pressure; SAP, systolic arterial pressure; Dev, development; Val, validation; CV, cross-validation.

**Table 2:** Model development characteristics.

Author/Year	No. of PIH events/Total sample size	Modeling methods (No. of models)	Predictor selection method	No. of predictors	Key predictors (examples)	Missing data handling
Lin 2011 (18)	482/1,311	ANN, LR (2)	Stepwise LR after clinical screening	5	Age, sex, emergency status, ASA, baseline SBP	Not reported
Kang 2020 (19)	126/222	NB, LR, RF, ANN (4)	RFE	23	a	Not reported
Li 2021 (20)	1,578/3,030	RF (1)	RF importance (MDG)	10	MAP, age, BMI, EF, ASA, IABP, diabetes, EuroSCORE I, creatinine, beta-blocker	Not reported
Yoshimura 2022 (21)	670/1,956	DNN, XGB, LDA, LR (4)	SHAP	49	b	Replaced with normal values
Zhao 2023 (22)	199/320	XGB + LR ensemble (2)	Correlation, RF importance, RFE, elastic net, hybrid	26	c	Correlation-based imputation
Park 2024 (23)	2,321/3,669	RF, LightGBM, AdaBoost, XGB, GBM, ARD (6)	Built-in feature importance	3	Mean DBP, minimum MAP, minimum DBP	KNN imputation
Noto 2024 (24)	93/161	LR (1)	ROC cut-off + multivariable LR	4	Baseline MAP, propofol, fentanyl, ketamine	Complete-case
Sheng 2024 (25)	192/318	LR, NB, KNN, SVM, NN, DT, XGB, RF (8)	Boruta	6	HUAC, age, L3 SMI, PNI, Hb, BMI	missForest
Wang 2024 (26)	82/260	LR, RF, SVM (3)	Univariate + multivariable LR	9	Age, BMI, ACEIs/ARBs, baseline HR/MAP, admission HR/MAP, HR change, MAP change	Model-based imputation
Yang 2024 (27)	211/407	LR (1)	Univariate + stepwise LR	5	Age, total cholesterol, sufentanil dose, admission MAP, HR decline	Not reported
Hashemi 2025 (28)	110/215	LR, SVM, KNN, DT, RF, GBM, XGB, LightGBM (8)	LDA/PCA/SVD + sequential selection	11	Age, ASA, sex, position, surgery type, losartan, early fluids, weight, HR, muscle relaxant, IV anesthetic	Not reported
Chen 2025 (29)	921/5,406	LR, RF, XGB, NN (4)	Variance filter, correlation, coefficient interpretation	10	Baseline DBP, age, sex, surgery type, baseline SBP, platelets, rocuronium, BUN, albumin, fentanyl	Median imputation
Sheng 2025 (30)	127/440	SVM (bECRIME-SVM) (15)	Metaheuristic (bECRIME) wrapper	7	Diabetes, drinking history, atropine, beta-blocker, total cholesterol, pre-induction BP	Mean imputation + SMOTE
Bian 2025 (31)	481/938	LR (1)	LASSO + multivariable LR	4	Cardiac dysfunction, ward baseline MAP, etomidate use, pre-induction MAP	Multiple imputation
Katsin 2025 (32)	4,948/20,309	RF, LR (2)	Information gain + SHAP	14	Propofol dose, pre-induction SAP/MAP, beta-blocker, hypertension, pre-induction HR, fentanyl, midazolam, rocuronium, age, height, weight, lidocaine, cardiac surgery	None (no missing)
Xu 2026 (33)	573/1,550	LR (1)	Univariate + backward LR	6	Surgical approach, position, maintenance anesthesia, stroke volume, pre-induction HR, pre-induction SBP	Complete-case
Zhu 2026 (34)	174/352	LR (1)	LASSO + forward LR	5	Pre-induction esmolol, pericardial effusion, etomidate-propofol, sufentanil dose, IVC diameter	Complete-case

ASA, American Society of Anesthesiologists; BMI, body mass index; HR, heart rate; IABP, intra-aortic balloon pump; ACEI, angiotensin-converting enzyme inhibitor; ARB, angiotensin receptor blocker; IVC, inferior vena cava; HUAC, Hounsfield unit average calculation; PNI, prognostic nutritional index; L3 SMI, L3 skeletal muscle index; RFE, recursive feature elimination; SHAP, Shapley additive explanations; SMOTE, synthetic minority over-sampling technique; LASSO, least absolute shrinkage and selection operator; LR, logistic regression; RF, random forest; LDA, linear discriminant analysis; PCA, principal component analysis; SVD, singular value decomposition; KNN, k-nearest neighbors.

^a^Kang 2020–23 predictors include age, baseline SBP/MAP/DBP, NIBP SBP/MAP/DBP min/max/mean/SD, propofol min/max/mean, remifentanil min/max/mean, plasma concentrations, effect-site concentrations, target concentrations, tidal volume mean, hypotension frequency/duration/average duration.

^b^Yoshimura 2022–49 predictors including ascending aorta diameter, transmitral A wave, heart rate, pulmonary venous S wave, tricuspid regurgitation pressure gradient, IVC expiratory diameter, fractional shortening, LV mass index, end-systolic volume, etc.

^c^Zhao 2023–26 predictors including age, sex, BMI, preoperative hypertension, baseline SBP/MAP/DBP, mean/min/delta of SBP/MAP/DBP, mean/min HR, shock index, etc.

### 3.3 Model development characteristics

Across the 17 studies, a total of 50 prediction models were developed, including 35 (70.0%) machine-learning models and 15 (30.0%) logistic regression models. Among machine-learning algorithms, random forest was the most frequently used (6 studies), followed by XGBoost (4 studies), support vector machine (3 studies), and artificial neural network (2 studies). Predictor selection methods varied: five studies used traditional univariate screening followed by multivariable logistic regression, while others applied modern approaches such as LASSO, random forest importance, SHAP, recursive feature elimination, Boruta, or a metaheuristic wrapper. The final number of predictors ranged from 3 to 49 (median 7). The most commonly included predictors were age (11 studies), baseline blood pressure (10 studies), propofol dose or concentration (5 studies), body mass index (4 studies), sex (4 studies), American Society of Anesthesiologists class (3 studies), and surgery type (3 studies). Missing data were present in 16 studies and were handled using various methods, including complete-case analysis, mean/median imputation, multiple imputation, missForest, k-nearest neighbors, model-based imputation, and correlation-based imputation. Only one study (Katsin et al.) reported no missing data. Detailed model development characteristics are presented in Table 2.

### 3.4 Model predictive performance

From each study, we selected the best-performing model based on the validation AUC (when available) or the training AUC (when no validation was reported), yielding 17 optimal models. Among these 17 models, logistic regression was the most frequent (7 models, 41.2%), followed by random forest (6, 35.3%), with other machine-learning algorithms (XGBoost, ensemble, deep neural network, bECRIME-SVM) constituting the remaining 4 (23.5%).

Regarding model performance, the reported AUC/C-statistics from the modeling groups for these 17 optimal models ranged from 0.68 to 0.95, while validation-set AUCs (internal or external) ranged from 0.654 to 0.893. Strictly defined external validation (different institution) was performed in only 1 study (33), yielding an AUC of 0.654. Temporal validation (same institution, different time period) was performed in 3 studies (18, 25, 31), with AUCs of 0.893, 1.000 (Sheng et al., discussed below), and 0.697, respectively. Calibration was assessed via calibration curves in 6 studies, the Hosmer-Lemeshow test in 6, and Brier scores in 3. Detailed performance metrics and model presentation are summarized in Table 3.

**Table 3:** Performance and presentation of the prediction models.

Author/Year	Performance discrimination	Performance calibration	Training AUC	Validation AUC	Validation method	Model presentation	Sensitivity (%)	Specificity (%)
Lin 2011 (18)	ANN: AUC 0.893	H-L test (*p* > 0.05)	—	0.893	Temporal validation (data from 2008)	ANN model	76.4	85.6
Kang 2020 (19)	RF: AUC 0.842 (1,000 × 4-fold CV)	Not reported	—	0.842	Repeated 1,000 × 4-fold CV	Machine learning model (RF)	83.7	—
Li 2021 (20)	RF: AUC 0.843 (test set)	Not reported	—	0.843	Random split (7:3)	Machine learning model (RF)	78.8	85.6
Yoshimura 2022 (21)	DNN: AUC 0.72 (test set)	Not reported	—	0.72	Random split (7:3), repeated 10×	DNN model	65	70
Zhao 2023 (22)	Ensemble: AUC 0.86 (LOOCV)	Not reported	—	0.86	LOOCV	XGB + LR ensemble model	84	79
Park 2024 (23)	RF/XGB: AUC 0.772 (test set)	Not reported	—	0.772	Random split (7:3)	Machine learning model (RF/XGB)	70.3	69.7
Noto 2024 (24)	LR: AUC 0.802 (full data)	Not reported	0.802	—	None (full data)	Regression equation	—	—
Sheng 2024 (25)	RF: AUC 1.000 (test set)	Calibration curve, Brier = 0.0766	—	1	Temporal validation (same institution, different time period)	Machine learning model (RF)	—	—
Wang 2024 (26)	LR: training AUC 0.95, validation AUC 0.93	Calibration curve, H-L test	0.95	0.93	Random split (7:3)	Nomogram	83	80
Yang 2024 (27)	LR: AUC 0.705 (full data)	Not reported	0.705	—	None (full data)	Regression equation	—	—
Hashemi 2025 (28)	RF: AUC 0.947 (CV)	Not reported	—	0.947	Unspecified CV	Machine learning model (RF)	85.5	—
Chen 2025 (29)	LR: AUC 0.74 (test set)	Brier 0.1291, calibration curve, DCA	—	0.74	Random split (7:3) + 5-fold CV	Regression equation (LR)	70	—
Sheng 2025 (30)	bECRIME-SVM: AUC 0.865 (10-fold CV)	Not reported	—	0.865	10-fold CV	bECRIME-SVM model	83.4	85.3
Bian 2025 (31)	LR: internal AUC 0.680, temporal AUC 0.697	H-L test, calibration curve, Brier = 0.223	0.68	0.697	Bootstrap (1000) + temporal validation	Nomogram and dynamic application	72	58
Katsin 2025 (32)	RF: AUC 0.732 (5-fold CV)	Not reported	—	0.732	5-fold CV	Machine learning model (RF)	76	—
Xu 2026 (33)	LR: internal AUC 0.701, external AUC 0.654	Calibration curve	0.701	0.654	Random split (7:3) + external validation (independent dataset)	Nomogram	—	—
Zhu 2026 (34)	LR: AUC 0.921 (full data, no independent validation)	H-L test (*p* = 0.111), calibration curve	0.921	—	None (full data)	Nomogram	—	—

CV, cross-validation; LOOCV, leave-one-out cross-validation; DCA, decision curve analysis; H-L, Hosmer-Lemeshow.

**Figure 2:**
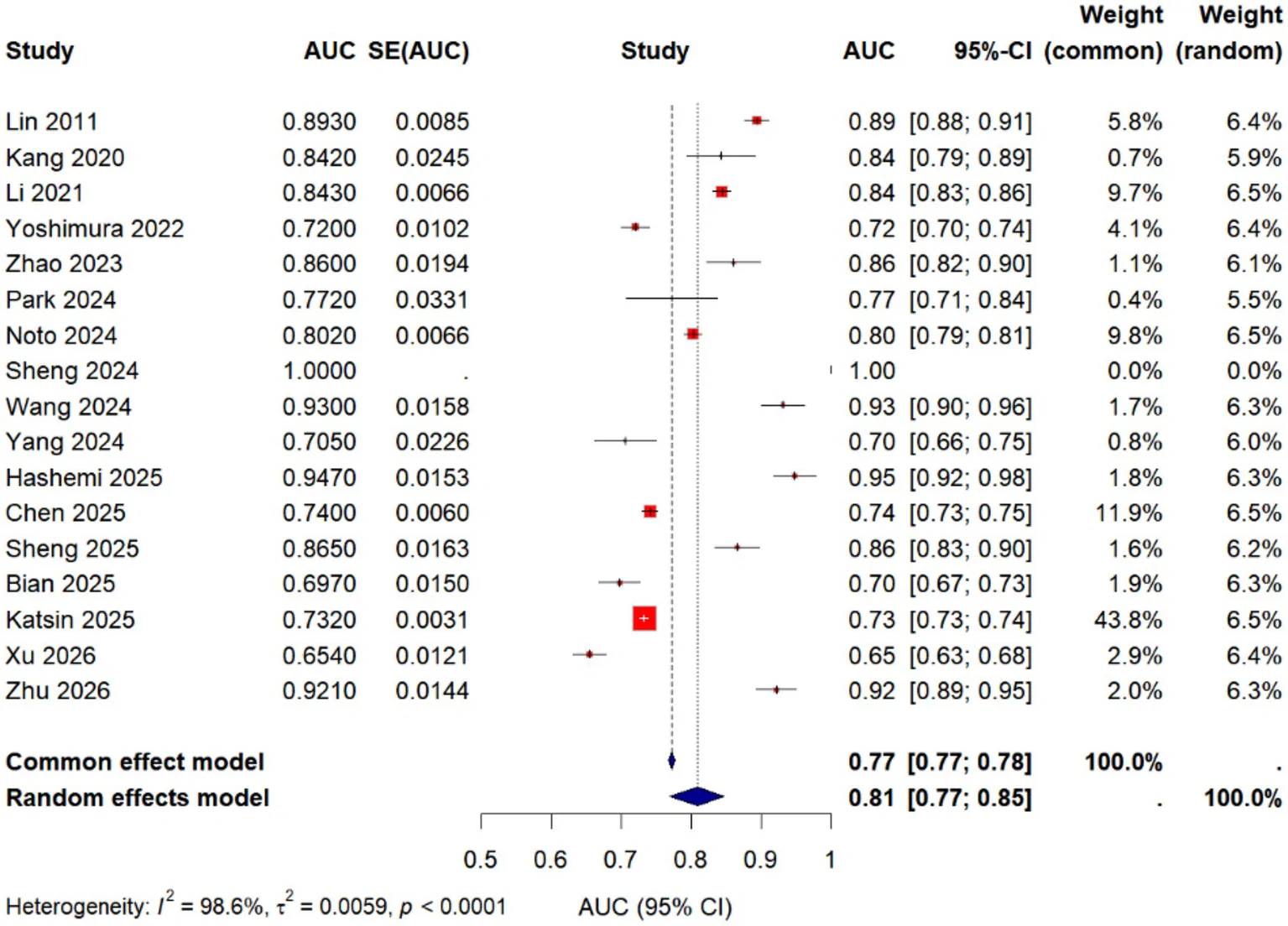
Forest plot of AUCs for PIH prediction models.

Pooled analysis using a random-effects model showed that the overall AUC of the 17 optimal models was 0.81 (95% CI 0.77–0.85), based on 3 model AUCs (from studies without validation) and 14 validation AUCs, with substantial heterogeneity (*I*^2^ = 98.6%; Figure 2). Sensitivity analysis excluding the four models with AUC > 0.9 yielded a pooled AUC of 0.78 (95% CI 0.74–0.82) for the remaining 13 models. As noted above, we also performed a sensitivity analysis restricted to the 14 validation AUCs only, which gave a pooled AUC of 0.81 (95% CI 0.76–0.85), confirming the robustness of our primary estimate.

Subgroup analyses (Table 4) revealed that the single multi-center study had an AUC of 0.95, whereas the 16 single-center studies had a pooled AUC of 0.80. Studies focusing on high-risk surgeries showed the highest pooled AUC (0.85), followed by mixed-risk surgeries (0.79). The pooled AUC was 0.84 for studies with sample size <1,000, compared with 0.78 for those ≥3,000. Models with <10 predictors and those ≥10 predictors both yielded 0.81. Regarding modeling methods, the pooled AUC was 0.82 for random forest, 0.79 for logistic regression, and 0.83 for other machine-learning methods.

**Table 4:** Subgroup meta-analysis of AUC.

Subgroup (n)		*I*^2^ (%)	Pooled AUC	95%CI	
				Lower	Upper
Overall (17)		98.6	0.81	0.77	0.85
After excluding extremes (13)		98.4	0.78	0.74	0.82
Validation (14)		98.7	0.81	0.76	0.85
Study design	Single-center (16)	98.5	0.80	0.76	0.84
	Multi-center (1)*	—	0.95	0.92	0.98
Surgical risk	High risk (3)	96.8	0.85	0.80	0.91
	Moderate risk (4)	99.0	0.83	0.66	1.00
	Mixed risk (10)	98.5	0.79	0.74	0.84
Sample size	<1,000 (10)	97.2	0.84	0.77	0.91
	1,000–3,000 (3)	99.2	0.77	0.64	0.90
	≥3,000 (4)	99.0	0.78	0.73	0.83
Event rate	<0.4 (7)	99.0	0.8	0.74	0.86
	0.4–0.6 (7)	97.4	0.82	0.75	0.89
	≥0.6 (3)	87.5	0.83	0.77	0.88
No. of predictors	<10 (10)	98.2	0.81	0.75	0.87
	≥10 (7)	98.7	0.81	0.76	0.86
Modeling method	LR (7)	97.0	0.79	0.70	0.87
	RF (6)	98.1	0.82	0.78	0.86
	Other (4)	91.5	0.83	0.76	0.91

*Only one study in this category; *I*^2^ not calculated.

It is important to highlight the extreme AUC value of 1.000 reported by Sheng et al. (25) in their temporal validation. Despite being a temporal validation, an AUC of exactly 1.0 is almost certainly a result of overfitting, likely due to the small validation sample size (*n* = 318), a large number of predictors (multiple CT-derived muscle parameters), and the use of complex algorithms without sufficient regularization. This perfect separation is biologically implausible and probably reflects data leakage or chance. We therefore treated this value as an extreme outlier in our sensitivity analysis and urge caution in interpreting this model’s performance.

A formal meta-analytic subgroup comparison between internally and externally validated models was not feasible due to the scarcity of true external validation (only one study). In descriptive terms, the single externally validated model (33) achieved an AUC of 0.654, which falls at the lower end of the range observed for temporally validated models (0.697–0.893) and for other internally validated models (0.68–0.95). This observation underscores the likelihood that performance estimates from internal validation may be optimistic, and highlights the urgent need for more external validation studies.

### 3.5 Meta-analysis of common predictors

Among the 17 included studies, ten did not provide usable effect size data for pooling (4 stated that data could not be shared, and 6 did not report detailed effect estimates). For the remaining 7 studies (18, 19, 24, 27, 31, 33, 34), predictors that appeared in at least 3 studies were pooled using a random-effects model (Table 5). Age was significantly associated with increased PIH risk (pooled OR, 1.031; 95% CI, 1.016–1.046; *p* < 0.001), with low heterogeneity (*I*^2^ = 9.8%). Propofol dose also showed a significant positive association (pooled OR, 1.357; 95% CI, 1.046–1.667; *p* < 0.001; *I*^2^ = 0%). Baseline MAP exhibited a direction suggesting a protective effect, but with very high heterogeneity (pooled OR, 0.961; 95% CI, 0.887–1.035; *I*^2^ = 95.2%), warranting cautious interpretation. Opioid dose showed a trend toward protection, but the association did not reach statistical significance (pooled OR, 0.659; 95% CI, 0.315–1.004; *I^2^* = 45.0%).

**Table 5:** Pooled effect sizes for common predictors.

Predictor	Pooled effect sizes				Heterogeneity	
	Pooled *OR*	95% *CI*	*Z* value	*p* value	*I*^2^ (%)	*p* value
Age	1.031	(1.016, 1.046)	137.860	< 0.001	9.769	0.330
Propofol	1.357	(1.046, 1.667)	8.565	< 0.001	0	0.506
Baseline MAP	0.961	(0.887, 1.035)	25.437	< 0.001	95.179	< 0.001
Opioids	0.659	(0.315, 1.004)	3.753	< 0.001	44.962	0.142

### 3.6 Risk of bias and applicability

Risk of bias and applicability were assessed using PROBAST (Table 6). Overall, 15 studies (88.2%) were rated as having high risk of bias, and 2 studies (11.8%) were rated as low risk of bias (29, 33). The primary sources of high risk of bias were concentrated in the analysis domain, including the use of univariate screening that may have missed important predictors, insufficient events per variable (EPV < 20), inappropriate or unreported handling of missing data, and poor calibration reporting (only 6 studies provided full calibration curves). Lack of external validation was also a concern: only 1 study (33) performed a true external validation on a different institutional dataset; the remaining 16 studies either used internal validation only or temporal validation, which we considered a form of internal validation. The remaining studies employed internal validation or no validation.

**Table 6:** PROBAST assessment of included studies.

Study	Risk of bias			Applicability				Overall risk of bias	Overall applicability
	Participants	Predictors	Outcome	Analysis	Participants	Predictors	Outcome		
Lin 2011 (18)	High	High	Low	High	High	High	High	High	High
Kang 2020 (19)	High	High	Low	High	High	High	Low	High	High
Li 2021 (20)	High	High	Low	High	High	High	Low	High	High
Yoshimura 2022 (21)	High	High	Low	High	High	High	Low	High	High
Zhao 2023 (22)	High	High	High	High	High	High	High	High	High
Park 2024 (23)	Low	Low	Low	High	Low	Low	Low	High	Low
Noto 2024 (24)	High	Low	High	High	High	High	High	High	High
Sheng 2024 (25)	High	Low	High	High	High	High	High	High	High
Wang 2024 (26)	High	Low	High	High	High	Low	Low	High	High
Yang 2024 (27)	High	High	High	High	High	High	High	High	High
Hashemi 2025 (28)	Low	Low	Low	High	Low	Low	Low	High	Low
Chen 2025 (29)	Low	Low	Low	Low	Low	Low	Low	Low	Low
Sheng 2025 (30)	High	High	Low	High	High	High	Low	High	High
Bian 2025 (31)	Low	Low	Low	High	Low	Low	Low	High	Low
Katsin 2025 (32)	High	High	Low	High	High	High	Low	High	High
Xu 2026 (33)	Low	Low	Low	Low	High	High	High	Low	High
Zhu 2026 (34)	High	High	High	High	High	High	High	High	High

For applicability, 4 studies (23, 28, 29, 31) were rated as low applicability concern. These studies enrolled broad non-cardiac surgical populations or mixed surgery types, used predictors that are routinely available in clinical practice (e.g., demographic data, baseline blood pressure, basic laboratory tests), and adopted PIH definitions aligned with common clinical thresholds. The remaining 13 studies had high applicability concerns, primarily due to highly selected participant groups (e.g., only elderly urological patients, acute type A aortic dissection, or colorectal cancer resection), use of specialized predictors not universally accessible (e.g., echocardiographic parameters, muscle CT-derived indices), or narrow PIH definitions that may not generalize to routine anesthesia practice. Consequently, the pooled AUC of 0.81 should be interpreted with caution, as model performance may differ when applied to broader or different clinical settings.

## 4 Discussion

Despite the well-recognized clinical significance of PIH, anesthesiologists currently lack accurate, individualized tools to preoperatively identify patients at highest risk, which limits the effectiveness of targeted preventive strategies (35). This gap in clinical decision support is particularly concerning given that PIH is largely preventable through appropriate interventions such as prophylactic vasopressors, optimized fluid therapy, and individualized anesthetic titration (36). To address this need, numerous prediction models have been developed in recent years, yet their methodological quality and generalizability remain uncertain. This systematic review and meta-analysis is the first to comprehensively evaluate PIH prediction models in patients undergoing general anesthesia, focusing on the best-performing model from each of the 17 included studies. Our pooled analysis, encompassing a total of 40,864 patients, yielded a summary AUC of 0.81 (95% CI 0.77–0.85), indicating good discriminative ability. The focus on the optimal model from each study provides a pragmatic estimate of the maximum predictive performance currently achievable in this field. Age and propofol dose were identified as the most consistent and robust predictors. However, our PROBAST assessment revealed that 88.2% of the included studies had a high risk of bias, primarily due to statistical analysis deficiencies, and a striking lack of external validation severely limits generalizability.

The pooled AUC of 0.81 in our study is comparable to that reported in meta-analyses of other perioperative prediction models, such as those for postoperative delirium (37) and deep venous thrombosis (38). This suggests that PIH is a predictable event when appropriate preoperative and intraoperative data are utilized. However, substantial heterogeneity (*I*^2^ = 98.6%) was observed across the included studies. This high heterogeneity is likely multifactorial, stemming from the considerable variations in PIH definitions, study populations, surgical types, and modeling techniques. For instance, the incidence of PIH varies dramatically depending on the threshold used and the time window considered (3). This definitional inconsistency is a major barrier to model comparison and clinical consensus. Subgroup analyses, while not completely eliminating heterogeneity, revealed that models developed for high-risk surgeries yielded a higher pooled AUC (0.85) than those for mixed-risk surgeries (0.79), likely because the patient population and hemodynamic insults are more pronounced and specific. Similarly, the pooled AUC for studies with a sample size of <1,000 was surprisingly higher than for those with ≥3,000 patients, which might be attributed to overfitting in smaller, more homogeneous, single-center datasets, underscoring the need for large, diverse populations for model development (39).

Our meta-analysis of predictors confirmed age and propofol dose as significant and robust risk factors for PIH. The pooled odds ratio for age (pooled OR 1.031) aligns with established knowledge that aging is associated with reduced vascular compliance, impaired baroreceptor sensitivity, and a higher prevalence of comorbidities, all of which compromise hemodynamic stability during anesthesia induction (40). The effect of propofol dose (pooled OR 1.357) provides a quantitative basis for the clinical recommendation to minimize propofol dosage, especially in high-risk patients. While propofol is a mainstay of induction, its dose-dependent vasodilatory and negative inotropic effects are primary drivers of PIH (41). Our findings support strategies such as propofol sparing using adjunctive agents or employing target-controlled infusion to achieve a more predictable effect-site concentration, as suggested by previous research (42). Interestingly, baseline MAP showed a protective trend but with high heterogeneity, likely because its predictive value is modulated by the patient’s autonomic nervous system function (43) and volume status. The protective trend observed for opioid dose, though not statistically significant, echoes the clinical observation that potent opioids like remifentanil can blunt the sympathetic response to laryngoscopy and intubation (44), though the net effect is highly context-dependent and requires more focused investigation.

The PROBAST assessment revealed a critically high risk of bias in the vast majority of included studies, with the analysis domain being the primary contributor. Specific issues included inappropriate handling of missing data, insufficient events per variable (EPV < 20), and reliance on univariate screening for predictor selection, which may miss important predictors that are only significant in a multivariable context (45). Additionally, many studies inadequately reported model calibration, with only a minority providing calibration curves or Hosmer-Lemeshow test results, despite the fact that good discrimination (AUC) does not guarantee good calibration and a poorly calibrated model can provide misleading risk estimates in clinical practice. A particularly critical gap is the striking lack of external validation: only one of the 17 included models underwent true external validation on a different institutional dataset, and its performance (AUC 0.654) was markedly lower than the pooled estimate of 0.81. While temporal validation at the same institution is a valuable step, it does not fully address concerns about overfitting to site-specific practices, patient mix, or data acquisition protocols (46). Due to the nature of systematic reviews, we cannot determine whether ongoing external validation studies exist for the included models; however, the absence of reported external validation in the published literature, coupled with the infeasibility of a formal subgroup analysis comparing internally versus externally validated models due to the scarcity of true external validation (*n* = 1), underscores that this is a major barrier to clinical translation. To translate these promising models into clinical practice, future research must prioritize methodological rigor and clinical utility. An urgent priority is the standardization of PIH definition, as recommended by the Perioperative Quality Initiative (POQI) (47), to harmonize research and enable meaningful comparisons. Model development should adhere to the TRIPOD statement (48), employing robust statistical methods such as multiple imputation for missing data and modern predictor selection techniques (e.g., LASSO) while ensuring adequate events per variable to prevent overfitting. Crucially, the field must pivot from repeatedly developing new models to prioritizing external validation, ideally in large, multicenter, prospective cohorts, to rigorously test generalizability before any clinical application. Finally, future studies must evaluate the clinical impact of these models by demonstrating that their use in decision support systems leads to improved processes of care and, ultimately, better patient outcomes, such as reduced PIH incidence and associated morbidity.

This review has several limitations that should be acknowledged. First, the high risk of bias in the included studies, as identified by PROBAST, directly impacts the confidence in our pooled estimates. The summary AUC of 0.81 may be an overestimation of true model performance. Second, substantial heterogeneity (*I*^2^ = 98.6%) persisted across studies, which, despite our subgroup analyses, could not be fully explained. Third, our meta-analysis of predictors was limited to only seven studies that provided sufficient data, and the results for some predictors, such as baseline MAP, were highly heterogeneous. Fourth, the search was restricted to English and Chinese databases, which may have led to the omission of relevant studies published in other languages. Finally, the long publication period (2011–2026) may reflect changes in clinical practice and data science techniques, making comparisons over time challenging.

## 5 Conclusion

In conclusion, current prediction models for PIH demonstrate good discriminative ability, with age and propofol dose emerging as the most significant and consistent predictors. However, the high risk of bias and striking lack of external validation severely limit the clinical applicability and generalizability of these models. Our findings underscore an urgent need for the field to shift its focus from developing new models to rigorously validating existing ones. Future work should adhere to methodological best practices, including standardizing the definition of PIH, using robust statistical methods, and, most importantly, conducting multicenter external validation and prospective impact studies to translate these promising tools into clinical practice to improve patient safety.

## Data Availability

The data that support the findings of this study are available from the corresponding author, Yuelai Yang, upon reasonable request.
