# Supplementary material for: Prediction models for post-induction hypotension in patients undergoing general anesthesia: a systematic review and meta-analysis
Source: Front Med (Lausanne). 2026 Sep 8;13:1887058. doi: 10.3389/fmed.2026.1887058 (PMC13593812; doi:10.3389/fmed.2026.1887058)
Supplement: Supplementary file 1 [file Data_Sheet_1.DOCX]

| Set# |  | Results |
| --- | --- | --- |
| 1 | ("Anesthesia, General") [MeSH Terms] OR ("General anesthe*" OR "General anaesthe*" OR "Anesthesia induction" OR "Anaesthesia induction" OR "Induction of anesthesia" OR "Induction of anaesthesia" OR "Post-induction" OR "Postinduction") [All Fields] | 131,604 |
| 2 | ("Hypotension") [MeSH Terms] OR ("Hypotension" OR "Low blood pressure") [All Fields] | 85,862 |
| 3 | ("Prediction Methods, Machine") [MeSH Terms] OR ("Predictive Learning Models") [MeSH Terms] OR ("Predictive model" OR "Prediction model" OR "prognostic model" OR "Nomogram" OR "Machine Learning") [All Fields] | 292,588 |
| 4 | #1 AND #2 AND #3 | 51 |

**Appendix S1.** Overall Search Strategy

PubMed

| Set# |  | Results |
| --- | --- | --- |
| 1 | ("General anesthesia" OR "General anaesthesia" OR "Anesthesia induction" OR "Anaesthesia induction" OR "Induction of anesthesia" OR "Induction of anaesthesia" OR "Post-induction" OR "Postinduction") in Title Abstract Keyword | 48111 |
| 2 | ("Hypotension" OR "Low blood pressure") in Title Abstract Keyword | 27734 |
| 3 | ("Predictive model" OR "Prediction model" OR "prognostic model" OR "Nomogram" OR "Machine Learning") in Title Abstract Keyword | 9312 |
| 4 | #1 AND #2 AND #3 | 11 |

Cochrane Library

EBSCO

| Set# |  | Results |
| --- | --- | --- |
| 1 | All Fields ("General anesthe*" OR "General anaesthe*" OR "Anesthesia induction" OR "Anaesthesia induction" OR "Induction of anesthesia" OR "Induction of anaesthesia" OR "Post-induction" OR "Postinduction") | 25,952 |
| 2 | All Fields ("Hypotension" OR "Low blood pressure") | 22,770 |
| 3 | All Fields ("Predictive model" OR "Prediction model" OR "prognostic model" OR "Nomogram" OR "Machine Learning") | 305,221 |
| 4 | #1 AND #2 AND #3 | 10 |

Embase

| Set# |  | Results |
| --- | --- | --- |
| 1 | ("General anesthesia" OR "General anaesthesia" OR "Anesthesia induction" OR "Anaesthesia induction" OR "Induction of anesthesia" OR "Induction of anaesthesia" OR "Post-induction" OR "Postinduction") :ti,ab,kw | 149,869 |
| 2 | ("Hypotension" OR "Low blood pressure") :ti,ab,kw | 119,053 |
| 3 | ("Predictive model" OR "Prediction model" OR "prognostic model" OR "Nomogram" OR "Machine Learning") :ti,ab,kw | 337,980 |
| 4 | #1 AND #2 AND #3 | 86 |

Web of Science

| Set# |  | Results |
| --- | --- | --- |
| 1 | "General anesthe*" OR "General anaesthe*" OR "Anesthesia induction" OR "Anaesthesia induction" OR "Induction of anesthesia" OR "Induction of anaesthesia" OR "Post-induction" OR "Postinduction" (Title) | 76,978 |
| 2 | "Hypotension" OR "Low blood pressure" (Title) | 67,524 |
| 3 | "Predictive model" OR "Prediction model" OR "prognostic model" OR "Nomogram" OR "Machine Learning" (Title) | 809,831 |
| 5 | #1 AND #2 AND #3 | 53 |

Chinese CNKI

| Set# |  | Results |
| --- | --- | --- |
| 1 | "Anesthesia induction" | 33,286 |
| 2 | "Hypotension" | 39,186 |
| 3 | "Prediction model" | 343,713 |
| 4 | #1 AND #2 AND #3 | 16 |

WanFang

| Set# |  | Results |
| --- | --- | --- |
| 1 | "Anesthesia induction" | 57,239 |
| 2 | "Hypotension" | 85,975 |
| 3 | "Prediction model" | 278,422 |
| 4 | #1 AND #2 AND #3 | 17 |
